# Endothelin-1 in combination with CRB-65 enhance risk stratification in COVID-19 patients

**DOI:** 10.1007/s15010-025-02627-4

**Published:** 2025-08-19

**Authors:** Imrana Farhat, Maciej Rosolowski, Katharina Ahrens, Jasmin Lienau, Peter Ahnert, Mathias Pletz, Gernot Rohde, Jan Rupp, Martin Witzenrath, Markus Scholz, CAPNETZ Study Group, PROGRESS Study Group

**Affiliations:** 1 Institute for Medical Informatics, Statistics, and Epidemiology (IMISE), University of Leipzig, Leipzig, Germany; 2 Department of Infectious Diseases, Respiratory Medicine and Critical Care, Charité – Universitätsmedizin Berlin, Corporate Member of Freie Universität Berlin and Humboldt-Universität zu Berlin, Berlin, Germany; 3 Associated Member of the German Center for Lung Research (DZL), Munich, Germany; 4 Institute of Infectious Diseases and Infection Control and Center for Sepsis Care and Control, Jena University Hospital/ Friedrich Schiller University Jena, Jena, Germany; 5 CAPNETZ STIFTUNG, Hannover, Germany; 6 Clinic for Pneumology, Intensive Care and Sleep Medicine, Universitätklinikum Giessen and Marburg GmbH, Marburg, Germany; 7 Institute of Medical Microbiology, University of Lübeck, University Hospital Schleswig-Holstein/ Campus Lübeck, Lübeck and Kiel, Lübeck, Germany

**Keywords:** COVID-19, CRB-65, Biomarkers, Risk prediction

## Abstract

**Background:**

COVID-19 continuously causes severe disease conditions and significant mortality. We evaluate whether easily accessible biomarkers can improve risk prediction of severe disease outcomes.

**Methods:**

Our study analysed 426 COVID-19 patients collected by German CAPNETZ and PROGRESS study groups between 2020 and 2021. Troponin T high-sensitive (TnT-hs), procalcitonin (PCT), N-terminal pro brain natriuretic peptide, angiopoietin-2, copeptin, endothelin-1 (ET-1) and lipocalin-2 were measured at enrolment and related to 28d mortality/ICU admission endpoint. Logistic and relaxed LASSO regression were used to evaluate the added value of biomarkers compared to the CRB-65 score and to develop a combined risk prediction model for our endpoint.

**Results:**

Of the 426 COVID-19 patients, 64 (15%) reached the endpoint. Among individual biomarkers, ET-1 showed the highest predictive performance (AUC = 0.76, 95% CI: 0.70–0.82). CRB-65 alone had an AUC of 0.63 (95% CI: 0.56–0.70). Our machine learning method identified CRB-65 + ET-1 to be optimal for prediction performance and model sparsity (AUC = 0.77, 95% CI: 0.71–0.83). Decision curve analysis demonstrated its greater net benefit over CRB-65 across large range of risk thresholds. The generalizability of our non-COVID CAP model (CRB-65 + TnT-hs + PCT) to COVID-19 patients was also assessed, yielding an AUC of 0.67 (95% CI: 0.60–0.74) for our primary endpoint. For 28d mortality alone as endpoint, it performed remarkably well (AUC = 0.90, 95% CI: 0.85–0.95).

**Conclusion:**

Combining the already established clinical CRB-65 score with ET-1 significantly improves risk prediction of intensive care requirement or death within 28 days in hospitalized COVID-19 patients.

**Supplementary Information:**

The online version contains supplementary material available at 10.1007/s15010-025-02627-4.

## Introduction

The high infectivity of the SARS-CoV-2 virus together with the high mortality rates of COVID-19 particularly in elderly or comorbid subjects induces a significant and continued global health burden [1]. Infected subjects suffer from fatigue, dry cough, high fever and often pulmonary disease [2]. Delayed intensive care unit (ICU) admission of infected patients significantly increases mortality rates. Therefore, precise risk evaluation and timely management of hospitalized COVID-19 patients is essential [3].

Researchers are actively working to improve risk stratification of COVID-19 patients and to modify the pneumonia scores that were originally developed for non-SARS-CoV-2 cases [4]. Recent studies examined, for example, the established CURB-65, CRB-65 and qSOFA as risk scores for the prognosis of patients with COVID-19 pneumonia. The CRB-65 score, which is the simplified version of CURB-65, incorporates four criteria: confusion, respiratory rate ≥ 30 breaths/min, blood pressure (systolic > 90 mmHg or diastolic ≤ 60 mmHg), and age ≥ 65 years. Patients with a CRB-65 score of 0 to 1 are categorized as the low-risk group, while those with a score greater than 1 are classified as high risk. The results indicate that 8.3% of patients are at high risk of mortality based on the qSOFA score (> 1 point), and 36.3% with CRB-65 score (> 1 point). These studies further show that the area under the curve (AUC) values for the CRB-65 score in predicting the need for intensive care are significantly higher than those for the qSOFA score [5–7].

In the present study, we investigated potential improvements of CRB-65 for COVID-19 patients using the following set of seven biomarkers also employed in improving the CRB-65 score for patients with non-COVID community acquired pneumonia (CAP) [8]: Previous studies showed that elevated levels of N-terminal pro B-type natriuretic peptide (NT-proBNP), a marker of cardiac stress and heart failure, and high sensitive troponin T (TnT-hs), an indicator of myocardial injury, are both strongly associated with mortality in COVID-19 patients [9–11]. These findings suggests that cardiac involvement and myocardial damage may play a significant role in the poor outcomes observed in severe COVID-19 cases. Procalcitonin (PCT), a marker typically used to indicate bacterial infection and systematic inflammation, has also been proposed as biomarker for prognosis and assessment of disease severity in COVID-19 patients [12, 13].

Endothelin-1 (ET-1) levels in hospitalized COVID-19 patients were higher during the acute phase of infection, suggesting endothelial dysfunction and a possible role in disease severity and vascular complications [14, 15]. Copeptin has been proposed as a biomarker to help distinguish COVID-19 pneumonia patients from those with community-acquired pneumonia (CAP) of other origins and has also shown potential for predicting severity in COVID-19 [16]. Lipocalin-2 has also been identified as potential biomarker associated with disease severity reflecting its involvement in inflammatory responses and tissue damage during both the acute and post-acute phases of the disease [17]. In contrast Angiopoietin-2 (Ang-2)may play a indirect role in COVID-19 through its effects on endothelial activation, vascular permeability, and regulation of blood pressure, contributing to vascular dysfunction and potentially worsening outcomes in severe cases [18].

Artificial intelligence, including machine learning methods, played a vital role in managing the COVID-19 pandemic, helping to explore available treatments and biomarkers for better treatments and risk stratification [8, 19–21]. In the present analysis, we investigated the biomarkers mentioned above regarding their potential to predict severe disease outcomes. Biomarkers were studied individually and in combination with the already established clinical score CRB-65 to improve risk prediction using a sophisticated machine learning model. We also evaluated the performance of a combined score of CRB-65 + TNT + PCT, which we proposed for non-COVID-19 CAP patients [8].

## Materials and methods

### Study population, data collection and endpoint

Data were collected by the PROGRESS [22] and CAPNETZ [23, 24] study groups, using a harmonized protocol (CAPNETZ project number 2020-06-02-FLUM). Patient enrolment and data collection by the PROGRESS and CAPNETZ study groups were approved by the ethics committees of Charité (approval number EA4/246/20) and Medizinische Hochschule Hannover (vote number 301–2008), respectively. 513 COVID-19 patients admitted to hospital were collected in the period between March 2020 and May 2022, covering infections with the original SARS-CoV-2 strain as well as the Alpha, Delta, and early Omicron (BA.1) variants. Most patients were infected during the pre-Omicron phases, with only a small subset potentially representing early Omicron cases. Later Omicron subvariants and the endemic phase of the pandemic were not represented in this cohort. The primary endpoint of our study was defined as a composite of ICU admission or death within 28 days of study inclusion. Patients with ICU admission at or before baseline were excluded (87 out of 513), resulting in 426 COVID-19 patients who were included in our analyses. Median age was 59 years, 228 patients (53.5%) were male and 198 (46.5%) were female. Seventeen patients (4%) died within 28 days of study inclusion, of whom 9 patients died at ICU. Fifty-six (13.2%) patients were admitted to ICU. Due to overlaps, a total of 64 (15%) patients reached our primary composite endpoint. Patient characteristics are given in Table 1.

**Table 1:** Comparison of baseline characteristics of COVID-19 patients

Characteristic	*N*	Overall, *N* = 426^1^	No EP, *N* = 362^1^	EP, *N* = 64^1^	*p*-value^2^
Age	426	59 (49, 71)	59 (47, 69)	66 (52, 78)	0.004
Sex (m/f)	426	228/198	187/175	41/23	0.067
Smoking (y/n)	406	133/273	105/240	28/33	0.018
COPD (y/n)	426	18/408	15/347	3/61	0.7
Comorbidities (y/n)	418	297/121	245/109	52/12	0.051
Respiratory/Pulmonary diseases (y/n)	426	73/353	63/299	10/54	0.7
Pre-existing heart failure (y/n)	426	19/407	17/345	2/62	0.8
Pneumonia associated confusion (y/n)	426	7/419	5/357	2/62	0.3
Autoimmune disease (y/n)	426	25/401	19/343	6/58	0.2
Neurologic disease (y/n)	426	14/412	11/351	3/61	0.5
Diabetes mellitus (y/n)	426	93/333	72/290	21/43	0.021
Malignant disease (y/n)	426	46/380	34/328	12/52	0.026

^1^ Median (IQR)

^2^ Wilcoxon rank sum test; Pearson’s Chi-squared test; Fisher’s exact test

### Biomarker measurements

Three of the biomarkers, i.e., TnT-hs, NT-proBNP and PCT, were measured at the laboratory Labor Berlin – Charité Vivantes GmbH using electrochemiluminescence immunoassays (ECLIA) provided by Roche Diagnostics. The other four biomarkers were assessed using ELISA kits for Ang-2 (R&D Systems), copeptin (Cloud-Clone Corp.), ET-1 (R&D Systems) and lipocalin-2 (RayBiotec). Some of the biomarker measurements were either below the limit of detection (LOD) or above the limit of quantification (LOQ) due to dilution factors applied in the initial assessments. These values were replaced by the respective limits. Descriptive statistics of biomarkers are presented in Table 2.

**Table 2:** Statistical overview of log-transformed biomarker measurements in the CAPNETZ and PROGRESS study cohorts. The values of LOD and LOQ are given without log transformation

	TnT-hs	NT-proBNP	PCT	Ang-2	Copeptin	ET-1	Lipocalin-2
Unit	ng/l	ng/l	ng/l	ng/ml	pg/ml	pg/ml	ng/ml
LOD	3	5	0.02	46.9	15.6	0.39	4.1
LOQ	-	70,000	100	3,000	1,000	25	1,000
Values below LOD	59	4	20	0	15	39	51
Values above LOQ	0	0	0	20	8	0	1
Missing values	5	5	6	0	1	0	0
Completeness rate	0.99	0.99	0.99	1	1	1	1
N (Total)	508	508	507	513	512	513	513
Mean	1.02	2.41	-0.95	0.68	2.24	0.43	0.86
Median	0.90	2.35	-1.05	0.57	2.27	0.48	0.84
SD	0.45	0.75	0.50	0.6	0.33	0.31	0.32
Min	0.48	0.70	-1.70	-0.35	1.19	-0.41	0.09

LOD: Lower Limit of Detection; LOQ: Upper Limit of Quantification

Measurements below LOD or above LOQ were replaced by the respective limits

### Statistical analysis

Statistical analyses were performed using log-transformed measurements of the biomarkers. Distributions of biomarkers were compared between patients reaching the endpoint and controls using the Mann-Whitney U-test. The association between biomarkers and the primary endpoint, as well as the added value of each biomarker in predicting the endpoint compared to CRB-65 was analysed using ROC curves and logistic regression models. We evaluated the area under the curve (AUC) of the ROC curves as a prediction performance measure and compared the AUCs of different models using Delong’s test, as implemented in the R-package pROC. Performance of differently complex logistic regression models were compared using likelihood ratio test.

We employed relaxed LASSO (least absolute shrinkage and selection operator) to develop a sparse prediction model. Missing values were imputed using the k-nearest neighbour method (R package “caret”, with the default number of neighbours equal to 5) [25]. The relaxed LASSO approach first selects relevant variables and then fits an ordinary least squares regression model to these selected variables [26, 27]. This relaxation step reduces the bias introduced by LASSO by unshrinking the regression coefficients when strong shrinkage is unnecessary, such as in high signal-to-noise ratio scenarios. This unshrinking can potentially improve the model’s prediction performance by allowing coefficients to take values closer to their unpenalized estimates while maintaining the variable selection benefits of LASSO [28]. We optimized the relaxation and regularization parameters using 10-fold cross-validation. Following a previously tested procedure [29], we selected the model with the fewest biomarkers whose log-likelihood loss did not exceed the minimum possible loss by more than 4%. The CRB-65 score was included in all models by not penalizing its coefficients. The model was trained on each training set and then applied to the corresponding test set. The final model was fitted to the complete dataset.

This model building process was internally validated using nested cross-validation to obtain honest estimates of the model’s discrimination (AUC), calibration, and clinical usefulness. First, the full data set (*n* = 426) was partitioned into 10 equally sized outer folds. We trained a model on nine of these folds and evaluated its performance on the remaining 10th fold. Training the model involved selecting values of the tuning parameters (relaxation and regularization parameters) by 10-fold cross-validation. For this purpose, we partitioned the training set into 10 equally sized inner folds. We trained models using several different values of the tuning parameters on each nine inner folds and evaluated the models on the remaining inner 10th fold, respectively. The values of the tuning parameters that resulted in the best performance (in terms of log-likelihood loss) averaged over the 10 folds were selected. Next, the model with the optimal values of the tuning parameters was applied to the 10th outer fold and the predicted probabilities of the outcome (EP) were estimated for these patients. The procedure was repeated for the 10 outer folds, resulting in predicted EP probabilities for all patients. These probabilities were then compared to the actual outcomes to assess the discrimination, calibration and clinical usefulness of the model building procedure.

**Fig. 1:**
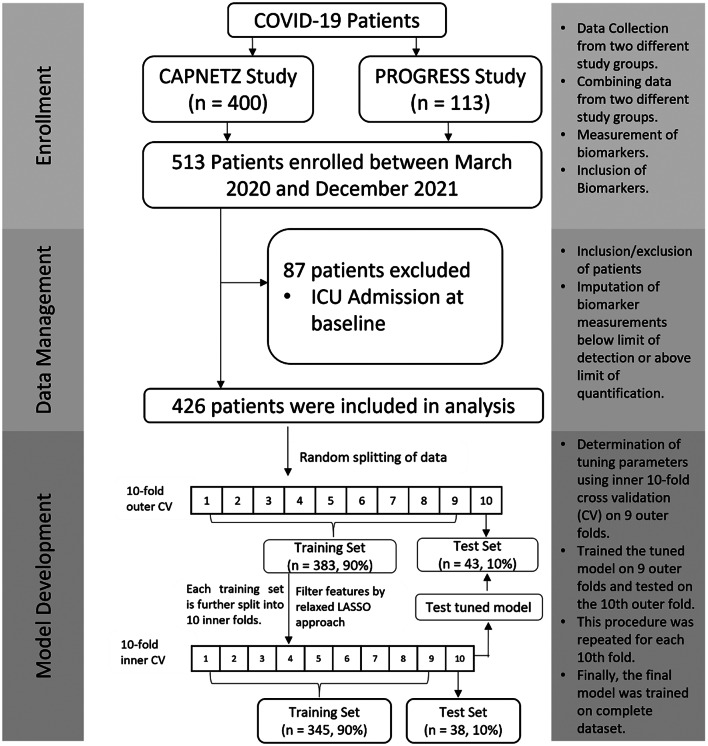
Study workflow: We describe the sampling of patients, the data management and the establishment of the statistical model. The latter is based on a two-fold cross-validation procedure to estimate both, the model parameters and the hyperparameters used for training

Figure 1 illustrates the workflow of patient selection and model development. Model development and reporting followed the TRIPOD guidelines [30, 31].

We also evaluated whether our recently proposed prediction model for non-COVID-19 CAP patients (combining CRB-65 with TnT-hs and PCT) remained predictive for COVID-19 patients. Clinical usefulness was assessed using decision curve analysis. All analyses were performed using R version 4.2.2.

## Results

### Predictive performance of CRB-65 combined with biomarkers

Patients who reached the composite endpoint showed significantly higher values of TnT-hs, NT-proBNP, ET-1, Ang-2, and PCT compared to those who did not (Fig. S1). In our primary analysis, the predictive performance for the composite endpoint of CRB-65 alone and in combination with individual biomarkers was assessed using logistic regression models and area under the receiver operating curves (AUC), see Table 3 and Fig. S2. CRB-65 alone achieved an AUC of 0.63 (95% CI: 0.56–0.70). The best single biomarker was ET-1 with an AUC of 0.76 (95% CI: 0.70–0.82), followed by PCT (AUC = 0.70, 95% CI: 0.60–0.74) and Ang-2 (AUC = 0.63, 95% CI: 0.56–0.70). Adding one of these three biomarkers to CRB-65 significantly improved prediction: ET-1 (AUC = 0.77, 95% CI: 0.72–0.83, p-value = 9.06e-11), PCT (AUC = 0.70, 95% CI: 0. 0.63–0.77, p-value = 9.90e-04), and Ang-2 (AUC = 0.67, 95% CI:0.59–0.75, p-value = 4.0e-3). Of note, ET-1 alone was not inferior to the model combining CRB-65 and ET-1 (*p* = 0.36).

**Table 3:** Comparison of prediction models of the primary endpoint “28d mortality or ICU admission”

	Area under ROC Curves		CRB-65 + Biomarker vs. Biomarker *p*-value	CRB-65 + Biomarker vs. CRB-65 *p*-value
	Biomarker alone	Biomarker + CRB-65		
TnT-hs	0.62	0.65	0.02	0.15
NT-proBNP	0.60	0.64	0.01	0.13
PCT	0.67	0.70	0.01	9.90e-4
Ang-2	0.63	0.67	0.05	4.5e-3
Copeptin	0.54	0.64	9.0e-4	0.75
ET-1	0.76	0.77	0.35	9.06e-11
Lipocalin-2	0.57	0.64	9.0e-4	0.46

AUC of CRB-65 alone is 0.63

**Fig. 2:**
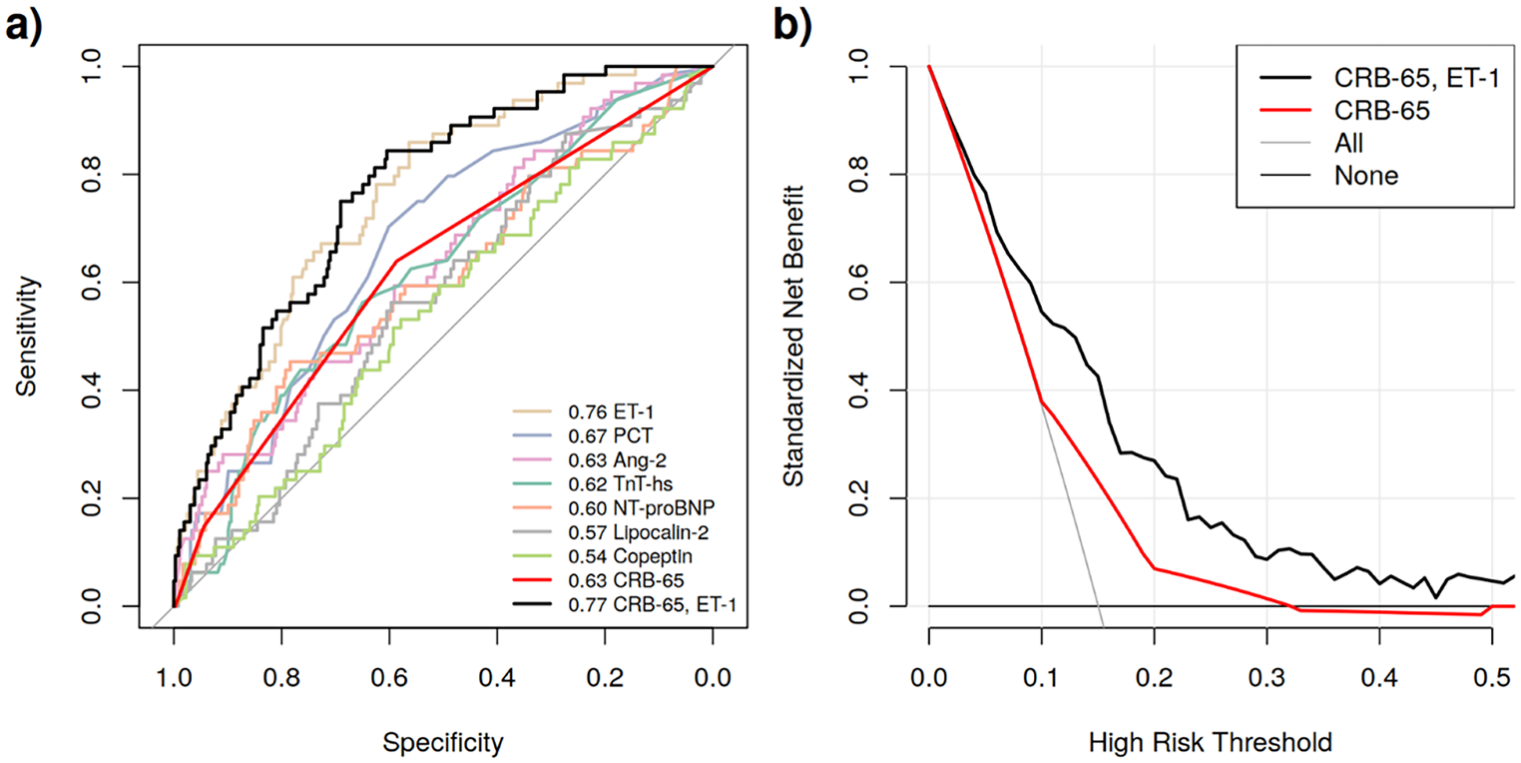
(**a**) Comparison of ROC curves of single biomarkers, CRB-65 alone and our selected prediction model (CRB-65 + ET-1). Respective AUCs are given in legends. (**b**) Decision curve analysis showing the standardized net benefit of our prediction model (black curve) and of CRB-65 alone (red curve) at different risk thresholds. Thresholds correspond to predicted probabilities of reaching the endpoint at which the clinician decides to treat the patient or to intervene in some other way

We further analysed whether combining multiple biomarkers with CRB-65 in a multivariable prediction model would result in additional improvement of risk prediction. Log-likelihood loss for different settings of the regularization parameters is shown in Fig. S3. Our relaxed LASSO method selected the combination of ET-1 and CRB-65 as the optimal model without adding other biomarkers. The multivariable model achieved an AUC of 0.77 (95% CI: 0.71–0.83) in nested cross-validation correcting for selection bias of factors, see Fig. 2a. An intercept of − 0.02 and a slope of 0.94 obtained in the calibration plot are both very close to the ideal values of 0 and 1, respectively, indicating good agreement between the predicted and observed risks (Fig. S4).

In a secondary analysis, we considered the two endpoints, 28d mortality and ICU admission, separately. The prediction model trained on the composite endpoint was evaluated for its performance against these secondary endpoints. For 28d mortality, an AUC of 0.82 (95% CI: 0.73–0.91) was achieved whereas for ICU admission, AUC was 0.75 (95% CI: 0.69–0.81).

### Clinical utility of the prediction model with CRB-65 and ET-1

Decision curve analysis assessed the clinical utility of our model combining CRB-65 and ET-1 compared to CRB-65 alone. The model demonstrated superior standardized net benefit across risk thresholds from 5 to 40% (Fig. 2b). For instance, at a threshold of 20%, corresponding to an acceptance of four false alerts for each true alert, our model achieved a standardized net benefit of 27%, which is equivalent to predicting 27% of patients requiring intensive care and no false positives (Table 4). In contrast, CRB-65 alone showed only 7% net benefit at this threshold. The net benefit of ET-1 alone at different thresholds is presented in Table S1.

**Table 4:** Numerical results of the decision curve analysis

Risk threshold	Cost-benefit ratio	TPR (sensitivity)	FPR (1-specificity)	PPV	NPV	Standardized net benefit
0.05	1:19	0.98	0.73	0.39	0.99	0.77
0.1	1:9	0.86	0.5	0.23	0.95	0.55
0.15	3:17	0.73	0.31	0.16	0.87	0.43
0.2	1:4	0.55	0.2	0.11	0.72	0.27
0.25	1:3	0.41	0.14	0.08	0.57	0.15
0.3	3:7	0.33	0.1	0.06	0.46	0.09
0.35	7:13	0.27	0.06	0.05	0.32	0.03
0.4	2:3	0.22	0.05	0.04	0.27	0.04

TPR: true positive rates, FPR: false positive rates, PPV: positive predicted values, NPV: negative predictive values and standardized net benefit are given for different risk thresholds

### Subgroup analysis

Our prediction model showed consistent performance across patient subgroups defined by age, sex, smoking status, diabetes mellitus, and other comorbidities (Fig. S5). The absence of significant AUC differences between subgroups suggests that the model performance is uniform across these risk factors.

ET-1 also maintained its predictive value within each CRB-65 risk group (Fig. S6). ROC curve analysis demonstrated comparable predictive performance of ET-1 across CRB-65 groups (*p* = 0.51, Fig. S7), supporting its value as an independent predictor regardless of the CRB-65 risk stratification.

### Risk Estimation formula

We propose the following formula for estimating the individual risk (probability) of the event, based on the parameters of our prediction model. This formula is based on a relaxed LASSO approach, which allows us to convert a combination of clinical and laboratory variables into a predicted probability ranging from 0 to 1. The risk estimation formula is given below:

$$\:\:\text{P}\text{r}\text{o}\text{b}\text{a}\text{b}\text{i}\text{l}\text{i}\text{t}\text{y}\:\text{o}\text{f}\:\text{t}\text{h}\text{e}\:\text{e}\text{v}\text{e}\text{n}\text{t}\:\left(p\right)=\frac{\text{exp}\left(x\right)}{1+\text{e}\text{x}\text{p}\left(x\right)},$$

where

$$\:x\:=\:-4.66\:+\:0.40\:*\:\text{C}\text{R}\text{B}65\:+\:4.82\:*\:\text{l}\text{n}\:\left(\text{E}\text{T}1\right).$$

Here, “ln” denotes the natural logarithm (logarithm to base e), exp(x) the exponential function, CRB65 the CRB-65 score and ET1 the ET-1 concentration in pg/ml. The coefficients of CRB-65 and ET-1 are standardized regression coefficients of the relaxed LASSO model, illustrating a relative contribution of parameters to the prediction of the outcome (EP). The risk estimation formula enables clinicians to determine a patient-specific probability of the event, thereby assisting in individual risk assessment and guiding management strategies.

### Validation of a prediction model developed for CAP patients

We evaluated whether our previously developed prediction model for non-COVID CAP patients (combining CRB-65, TnT-hs, and PCT) generalizes to COVID-19 patients [8]. For our primary composite endpoint, this model achieved an AUC of only 0.67 (95% CI: 0.60–0.74). For our secondary endpoint “admission to ICU” the model achieved an AUC of 0.63 (95% CI: 0.55–0.70). However, for 28-day mortality, it showed excellent discrimination with an AUC of 0.90 (95% CI: 0.85–0.95), comparable to its performance in CAP patients (Fig. S8).

We also compared the performance of both prediction models for secondary endpoints in non-COVID CAP patients and those with COVID-19. Both models have shown similar performance for predicting 28d mortality alone (Table S2, Fig. S9 and Fig. S10). The effect of SARS-CoV-2 vaccination on prediction performance was also evaluated. A total of 15.5% of our COVID-19 patients were vaccinated. The CRB-65 and ET-1 model achieved an AUC of 0.78 (95% CI: 0.71–0.84) in unvaccinated patients and 0.72 (95% CI: 0.57–0.88) in vaccinated patients (*p* = 0.55; Fig. S11) suggesting similar performance of the score in vaccinated and unvaccinated subjects.

## Discussion

Severity of COVID-19, caused by SARS-CoV-2, ranges from mild symptoms to potentially life-threatening complications. Despite employing a variety of diagnostic methods [32], the development of risk prediction models for the assessment of disease severity is still crucial for timely disease management. For the development of a multivariate prediction model for risk stratification of COVID-19 patients, the established clinical score CRB-65 and biomarkers TnThs, PCT, ET-1, Ang-2, NT-proBNP, copeptin and lipocalin-2 were considered. We examined the effectiveness of these serum marker levels in predicting outcomes for COVID-19 patients using both univariate and multivariate logistic regression models against the endpoint “28d mortality or ICU admission”. Our machine learning approach identified the combination of the biomarker ET-1 and CRB-65 as the best performing model (AUC = 0.77; 95% CI: 0.71–0.83). We demonstrated that the addition of ET-1 significantly improved the predictive performance of CRB-65. In contrast, adding CRB-65 to ET-1 did not result in a relevant improvement and could be omitted for our patient sample. However, since our study was specifically designed to enhance the predictive capacity of the widely used and well-established CRB-65 score, we are cautious about recommending its exclusion based on the findings of our relatively small study.

ET-1 also shows promise as a prognostic biomarker in COVID-19 ARDS, as higher systemic arterial-to-venous (A: V) ET-1 ratios upon ICU admission were significantly associated with 28-day mortality. This finding indicates that early disruption of pulmonary ET-1 clearance identify COVID-19 patients at higher risk, highlighting ET-1’s potential both as a marker and as a therapeutic target [33]. Similarly, another study has demonstrated that higher ET-1 levels were strongly associated with increases 30-day mortality in COVID-19 patients, highlighting its role as a marker of early disease severity [34]. In addition, studies have shown that the patients with severe COVID-19 exhibit elevated levels of autoantibodies against the endothelin-1 type A receptor (ETAR), which mimic ET-1 and cause sustained vasoconstriction and inflammation [35].

Since 28-day mortality is low (4%), we opted for our composite endpoint in primary analysis. This endpoint proved to be useful for similar research questions as well [36]. However, we observed a better prediction performance for mortality compared to ICU admission. Using the same endpoint and considering the same biomarkers, in a previous study, we developed a prediction model for non-COVID-19 CAP which consists of CRB-65 combined with TnT-hs and PCT [8]. Applied to COVID-19 patients, this model resulted in an inferior AUC for the composite endpoint compared to the CRB-65 + ET-1 model proposed here. However, for the endpoint “28d mortality”, the model showed a remarkably high predictive performance.

The distinct predictive utility of biomarkers for ICU treatment and mortality in non-COVID-19 CAP and COVID-19 observed in our analyses might possibly reflect differences in their underlying pathophysiology. For predicting the need of ICU treatment only, TnT-hs and PCT could be better suited for non-COVID CAP [37], as they tend to capture cardiac stress and bacterial inflammation, while ET-1 might be more effective in COVID-19 due to its association with endothelial dysfunction caused e.g. by pulmonary endothelitis [38], pulmonary vasoconstriction, inflammation and thrombosis, which are a hallmark of COVID-19 [39]. On the other hand, for predicting mortality alone, in both diseases the models with CRB65 + TnT-hs + PCT and CRB65 + ET-1 showed similar performances. This may be due to their ability to reflect systemic physiological derangements, such as cardiac injury and inflammatory dysregulation, which could be central to poor outcomes regardless of the specific infectious disease [38, 40, 41].

Our study has several limitations that should be considered when interpreting the findings. First, our sample size of 426 COVID-19 patients may not fully capture the variation present in the broader population, including differences in ethnicities, lifestyles, and pre-existing conditions, which could influence biomarker levels and disease outcomes [42–44]. Additionally, the limited number of cases with severe outcome in our cohort may affect the generalizability of our results. Thus, validation of our prediction model in larger cohorts representing a broader spectrum of risk factors is required. Furthermore, the samples were collected during the early phases of the pandemic in 2020 and 2021. Accordingly, 84.5% of our patients were unvaccinated. On the other hand, we did not observe a significant difference in prediction performance between vaccinated and unvaccinated patients (*p* = 0.55 of AUC comparison. While the temporal context might limit direct translation to current endemic conditions with different SARS-CoV-2 variants and established immunological memory, our study points out potential mechanistic insights for a well-defined patient cohort, raising the hypothesis that similar mechanisms may apply to the current endemic COVID-19 situation and possibly to virus induced CAP in general. Future studies are needed to explore potential broader applicability of our findings. Regarding translation of our findings into clinical routine, the primary challenge lies in the current lack of standardized and widely available assays for ET-1 measurement. While technical solutions such as mass spectrometry–based methods could enable accurate quantification in the future, the use of more stable surrogate markers like big endothelin-1 already measurable with commercial assays may serve as a pragmatic interim step toward clinical implementation.

## Conclusion

Our data suggest that combining the already established clinical CRB-65 score with ET-1 significantly improves risk prediction in hospitalized COVID-19 patients regarding the composite endpoint “admission to intensive care unit or death within 28 days”. Further studies are required to validate this finding under endemic conditions of COVID-19, to evaluate the relevance for viral CAP in general and to show benefit for patients in clinical practice.

## Supplementary Information

Below is the link to the electronic supplementary material.

Supplementary Material 1

## Data Availability

No datasets were generated or analysed during the current study.
