# Supplementary material for: Endothelin-1 in combination with CRB-65 enhance risk stratification in COVID-19 patients
Source: Infection. 2025 Aug 19;53(6):2769–78. doi: 10.1007/s15010-025-02627-4 (PMC12675736; doi:10.1007/s15010-025-02627-4)
Supplement: Supplementary file 1 — Supplementary Material 1 [file 15010_2025_2627_MOESM1_ESM.docx]

**Endothelin-1 in Combination with CRB-65 Enhance Risk Stratification in COVID-19 Patients**

**Supplementary Figures**

**Supplementary Tables**

**
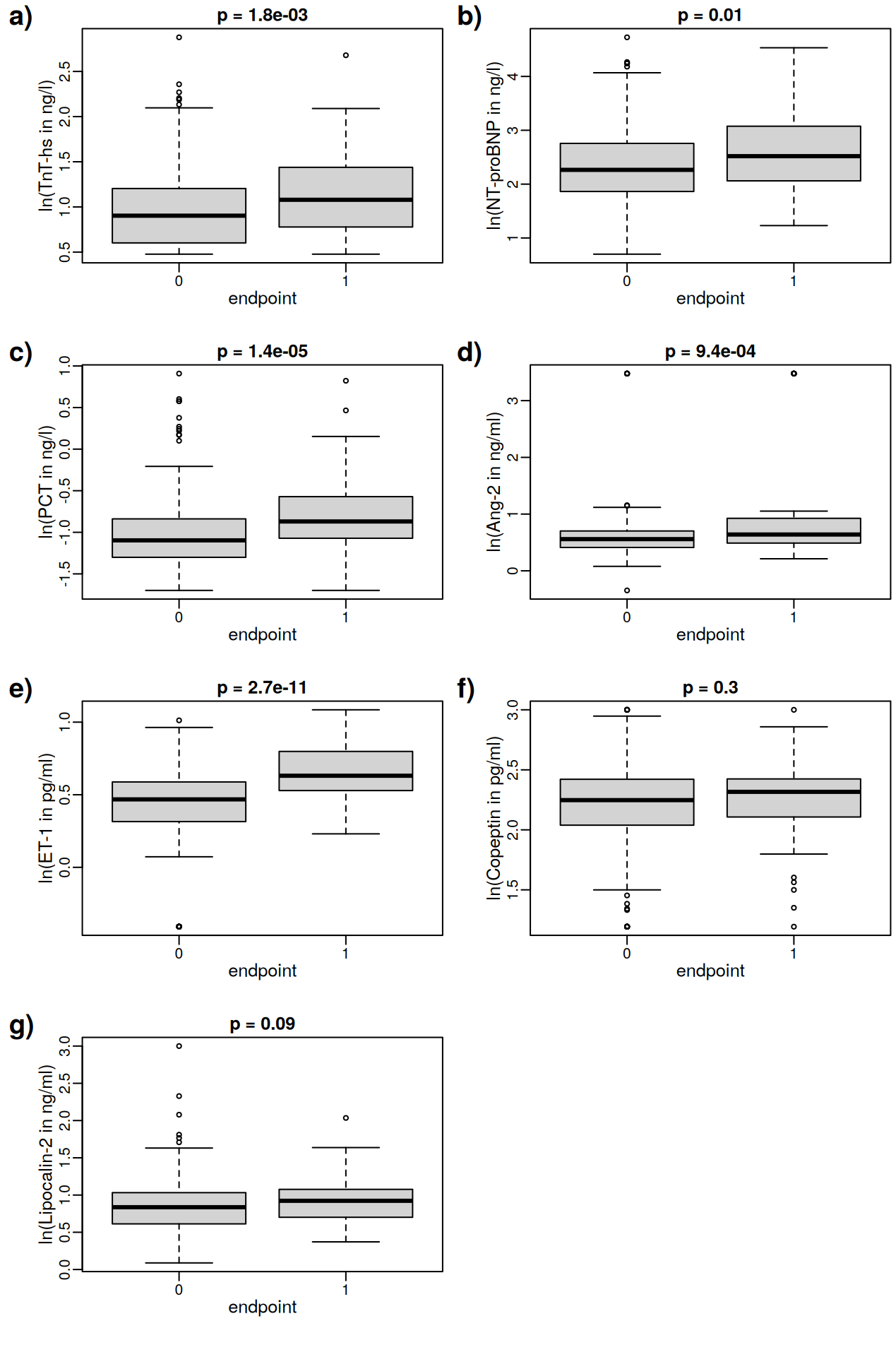
Supplementary figures:**

**Fig. S1:** Box plots illustrates the log-transformed distributions of biomarkers for cases and controls with respect to the primary endpoint. Significant differences were observed for all biomarkers except for copeptin and lipocalin-2. Statistical comparisons between patients who reached the endpoint and controls were conducted using Mann-Whitney U-test.


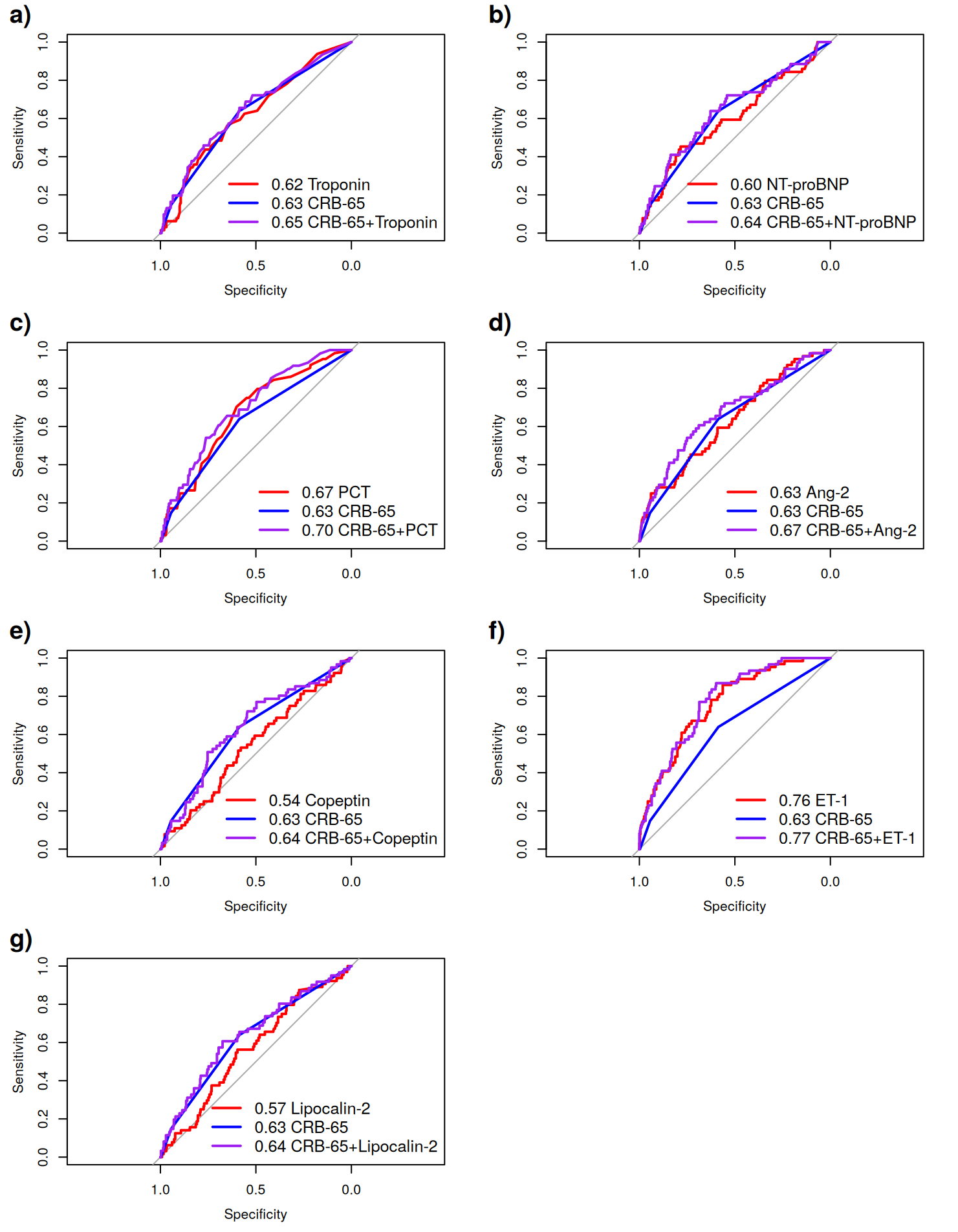


**Fig. S2:** Comparison of ROC curves for CRB-65, single biomarkers and the combination of CRB-65 and single biomarkers. We present AUCs in the legends. Note that CRB-65 is a categorical variable, while the biomarkers are continuous, which explains the smoother appearance of the ROC curve for CRB-65.


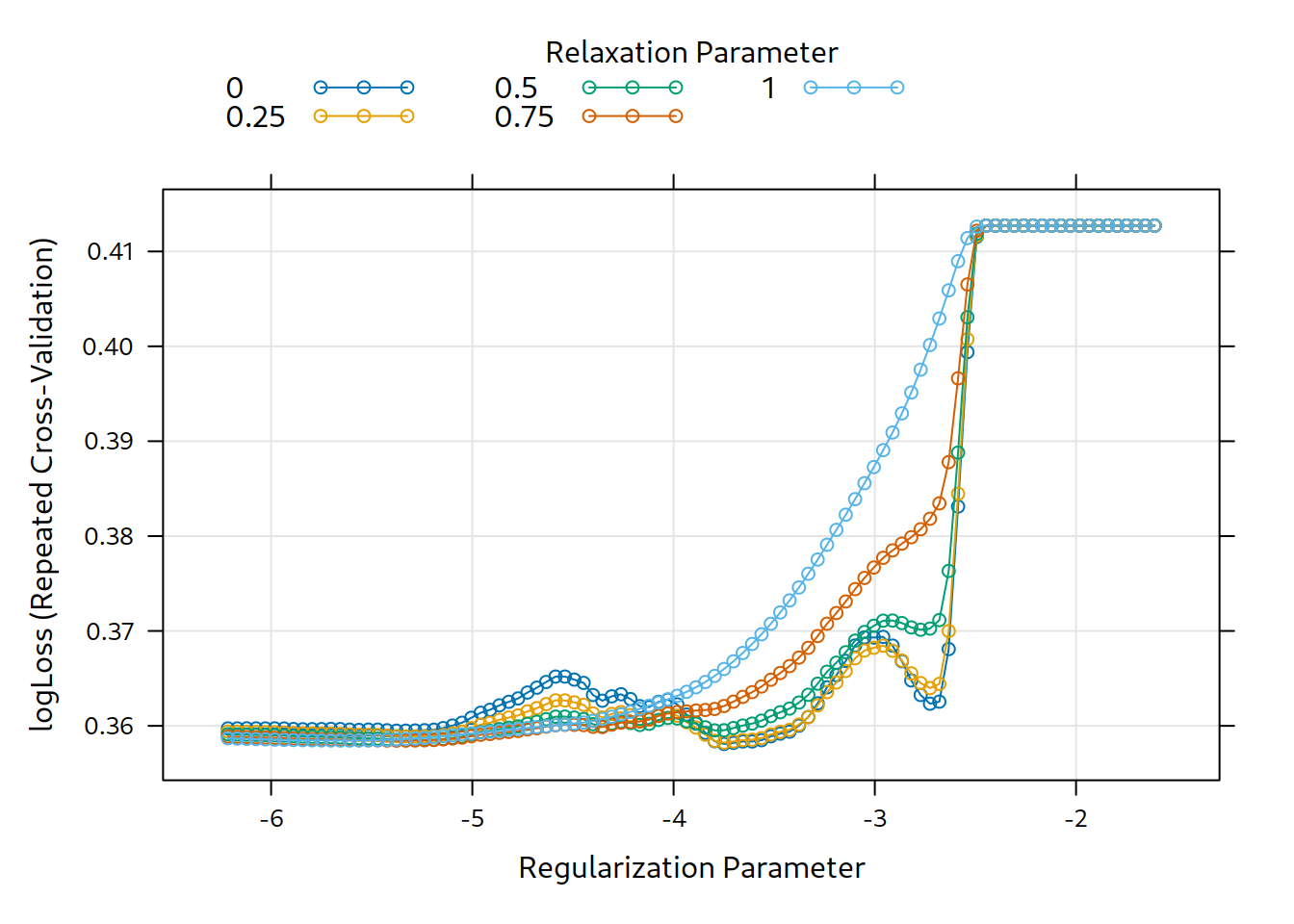


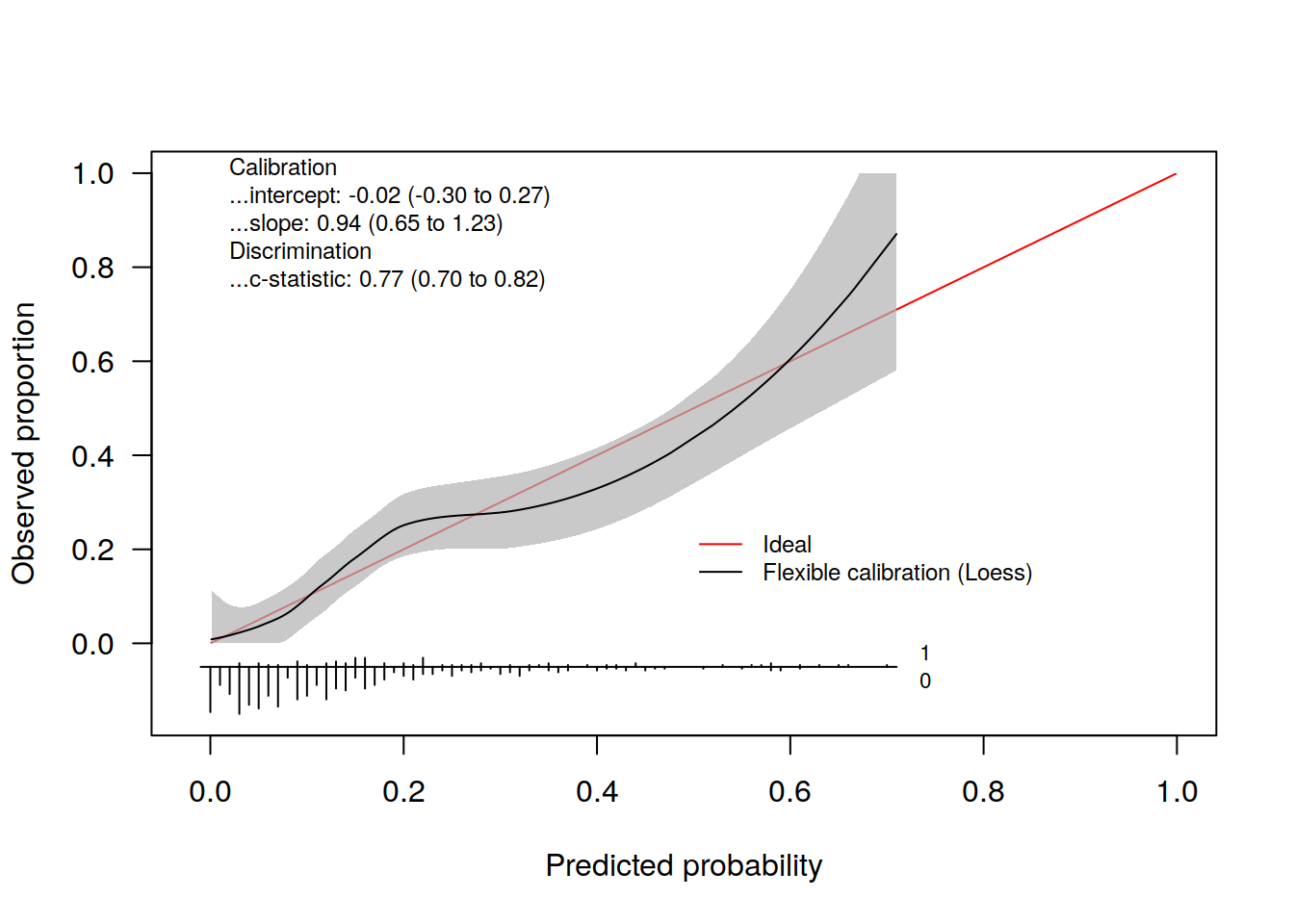
**Fig. S3:**  Comparison of regularization (x-axis) and relaxation parameter settings. The relaxation parameter equal to 0 (dark blue curve overlaps the yellow curve with relaxation parameter equal to 0.25) is optimal, combining low log loss and high model parsimony.

**Figure S4:** Cross-validated calibration curve. The predicted probabilities were obtained using nested cross-validation, i.e., they were predicted in each 10^th^ fold of the data set using a model trained and optimized on the remaining 9 folds, to ensure unbiased prediction. The rug plot shows the distribution of the predicted risks and grey area indicates the confidence band of the calibration curve. We obtained a calibration curve intercept of -0.02 and a slope of 0.94 indicating good model calibration according to (van Calster et al., 2019)


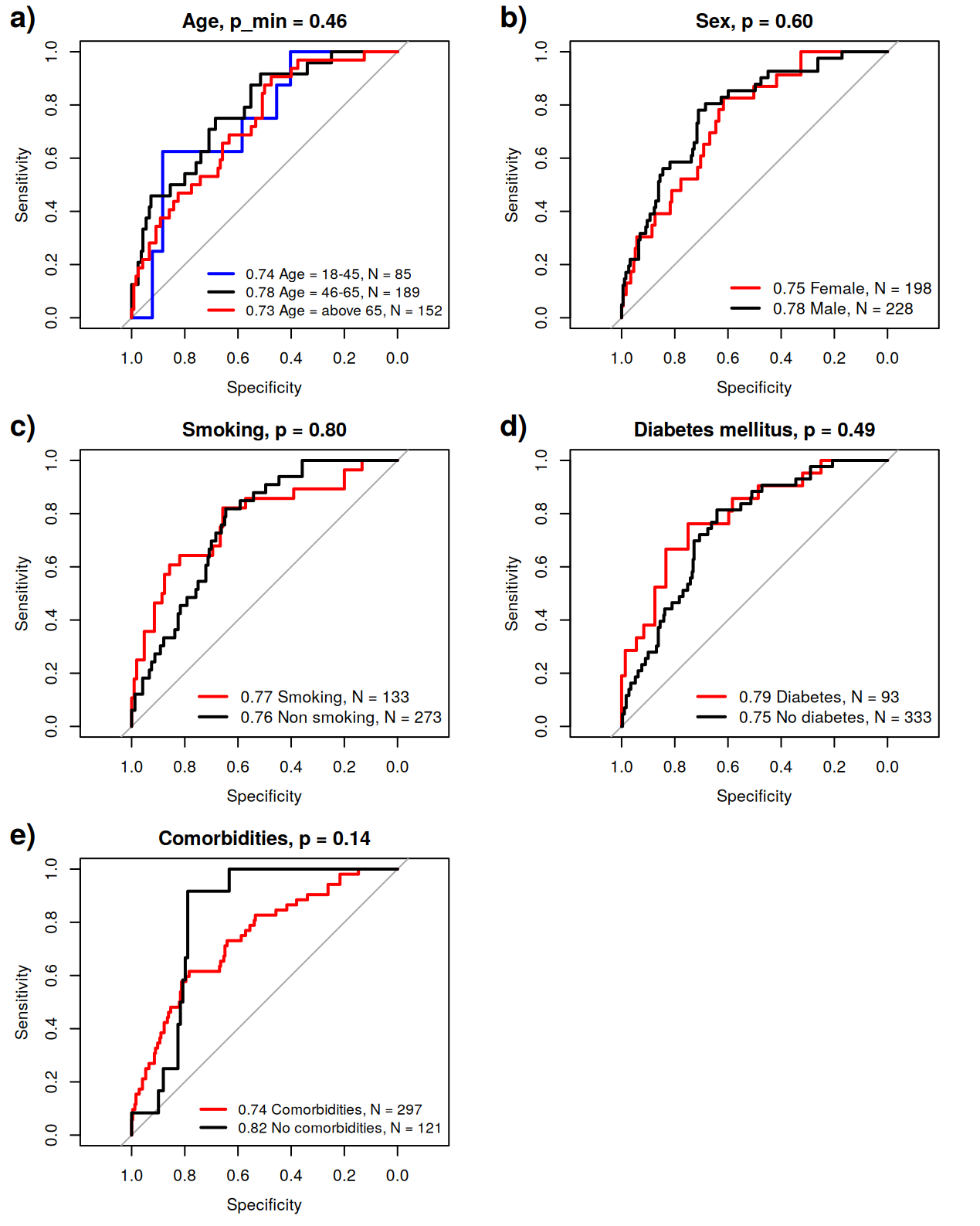
**Fig. S5:** Comparison of prediction performances across subgroups. For risk factor age, minimum p-value of the three pairwise group comparisons is given. For comorbidities, the number of patients without comorbidities who reached the primary endpoint was relatively low, resulting in fewer data points and a smoother appearance of the corresponding curve (black curve for “No comorbidities”).


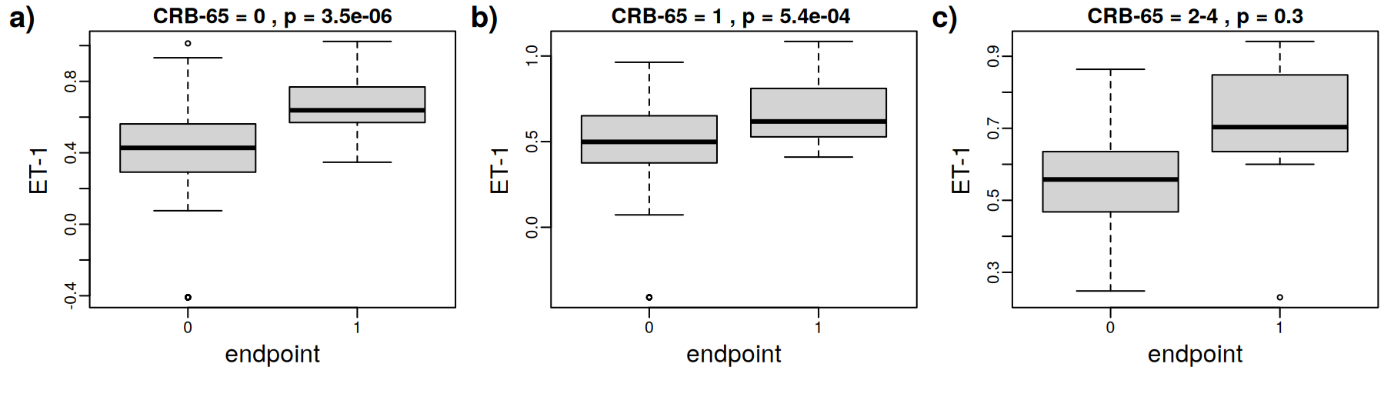


**Fig. S6:** ET-1 levels were compared between EP cases vs. controls within the different risk groups of the CRB-65 score. Due to small sample size, CRB-65 groups 2,3 and 4 were pooled. ET-1 always showed higher levels in cases compared to controls.


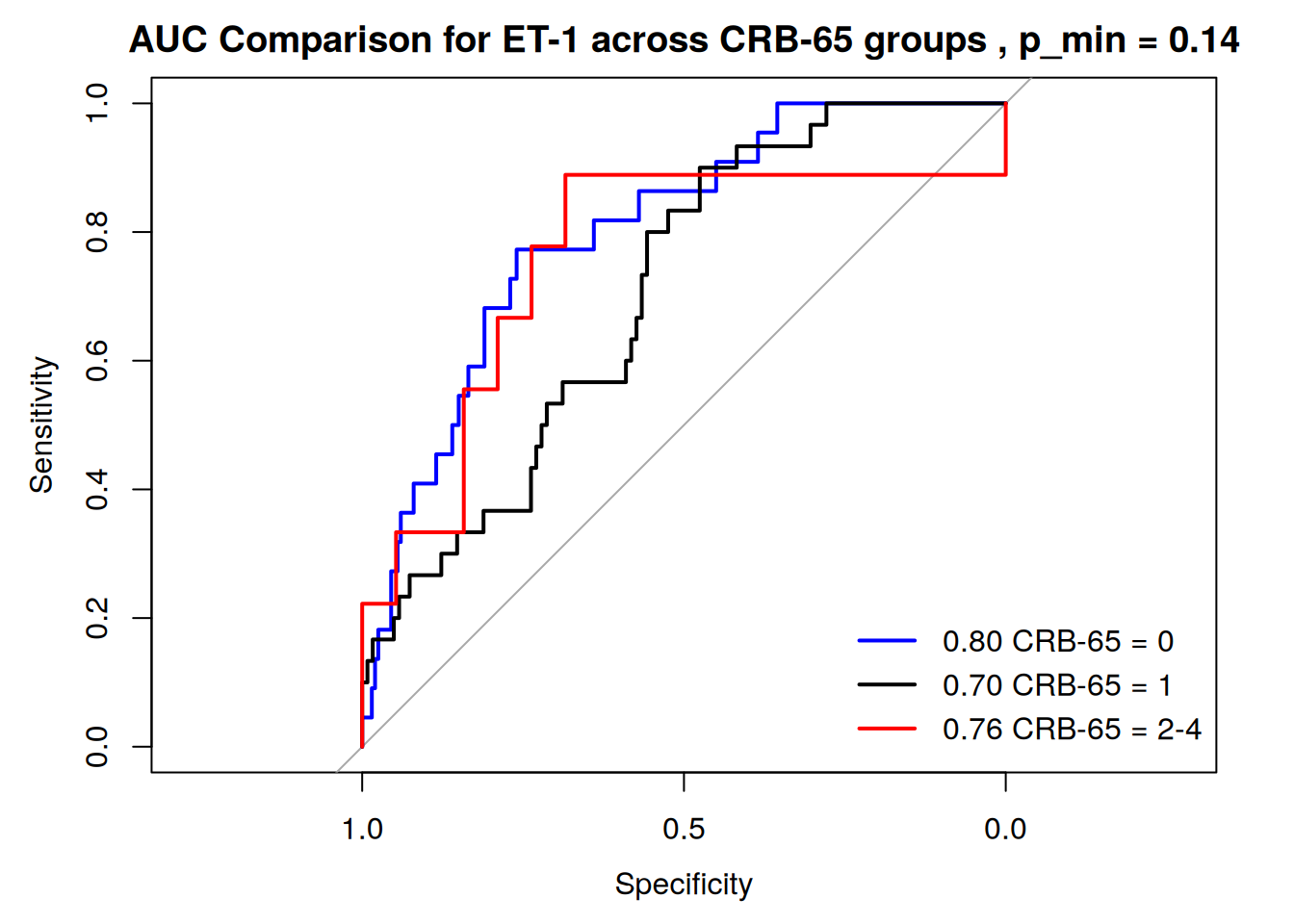


**Fig. S7:** Comparison of ROC curves of ET-1 between different CRB-65 risk groups. No significant differences were observed suggesting uniform prediction potential of ET-1. The minimum p-value of the three pairwise group comparisons is also given.


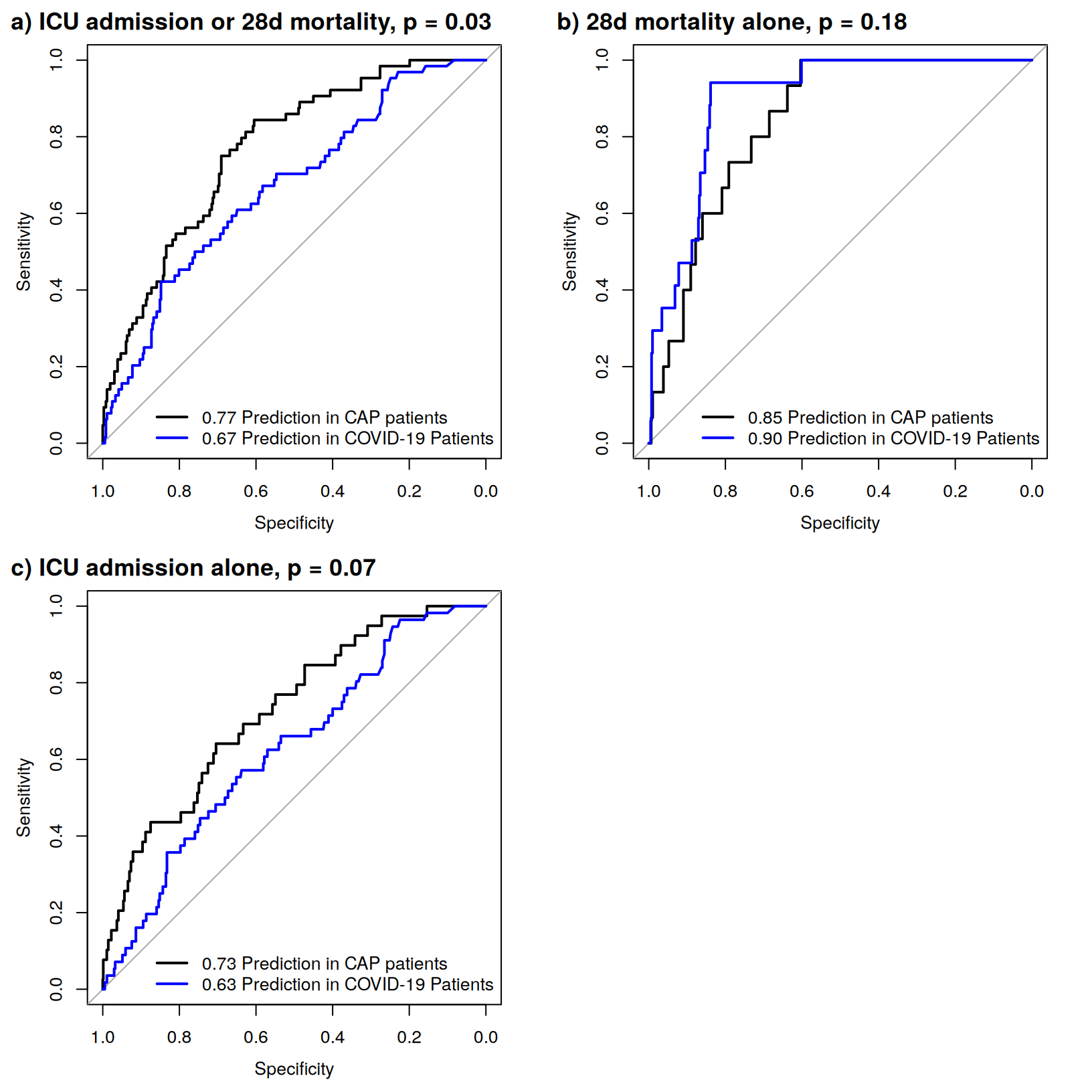
**Fig. S8:** Comparison of ROC curves to compare the prediction performance of CAP model in CAP patients with the validation results of the CAP model in COVID-19 patients against the three endpoints **a)** ICU admission or 28d mortality, **b)** 28d mortality alone, and **c)** ICU admission alone.


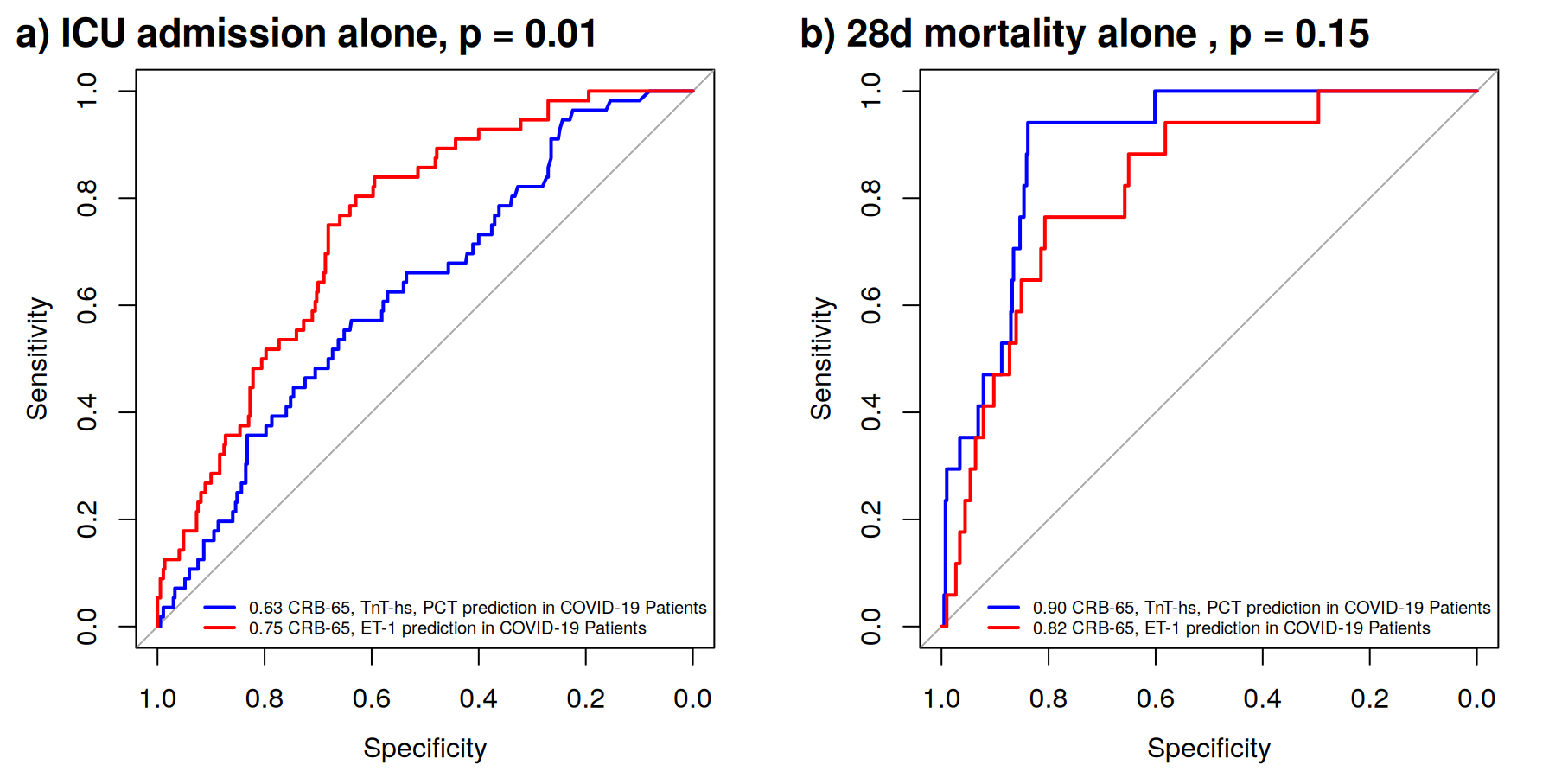


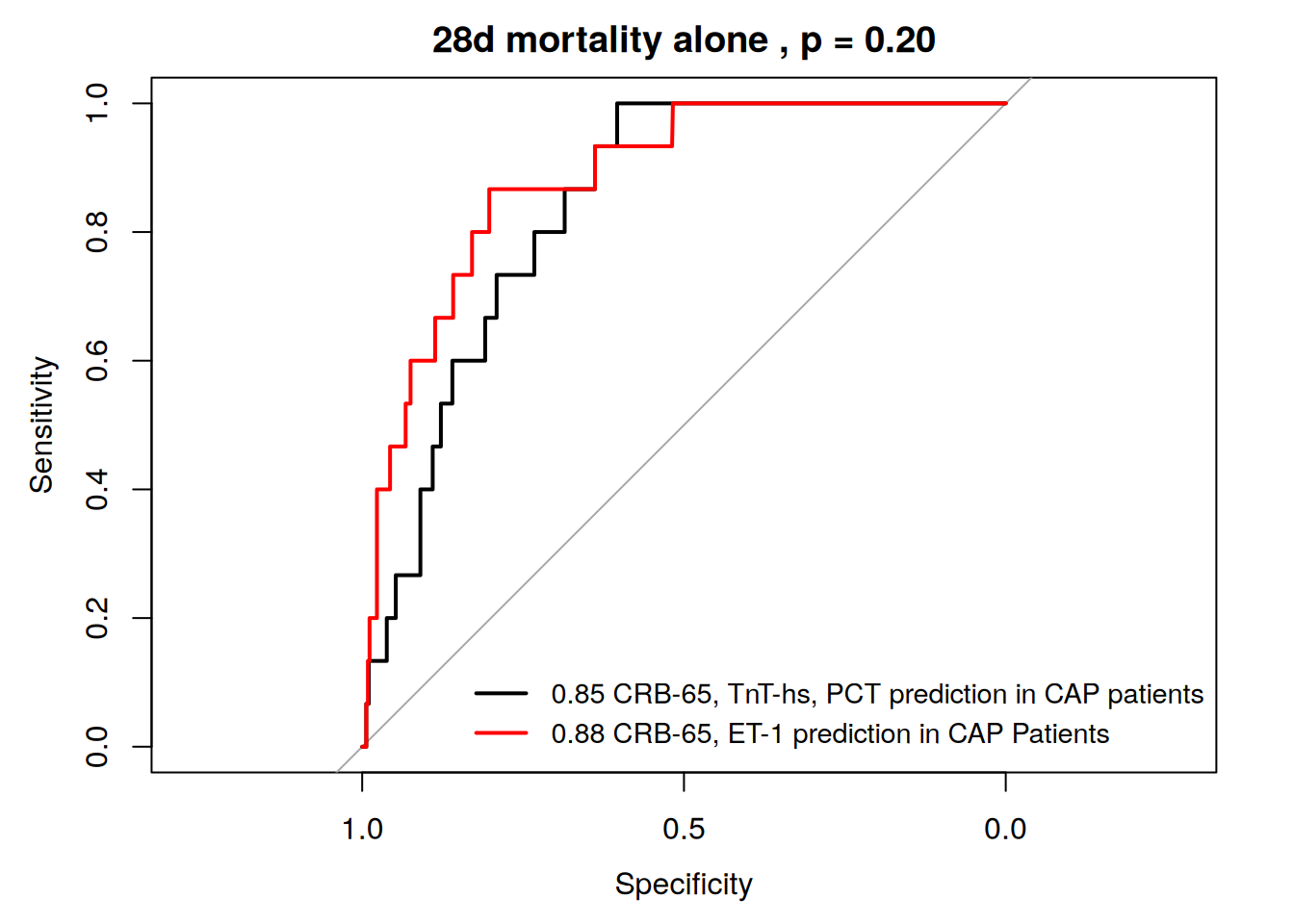
**Fig. S9:** Comparison of CRB-65, TnT-hs and PCT (CAP prediction model) with CRB-65 and ET-1 (COVID prediction model) to assess the performance of these models in predicting ICU admission alone and 28d mortality alone in COVID-19 patients.

**Fig. S10:** Comparison of CRB-65, TnT-hs and PCT (CAP prediction model) with CRB-65 and ET-1 (COVID prediction model) to assess the performance of these models in predicting 28d mortality alone in CAP patients.


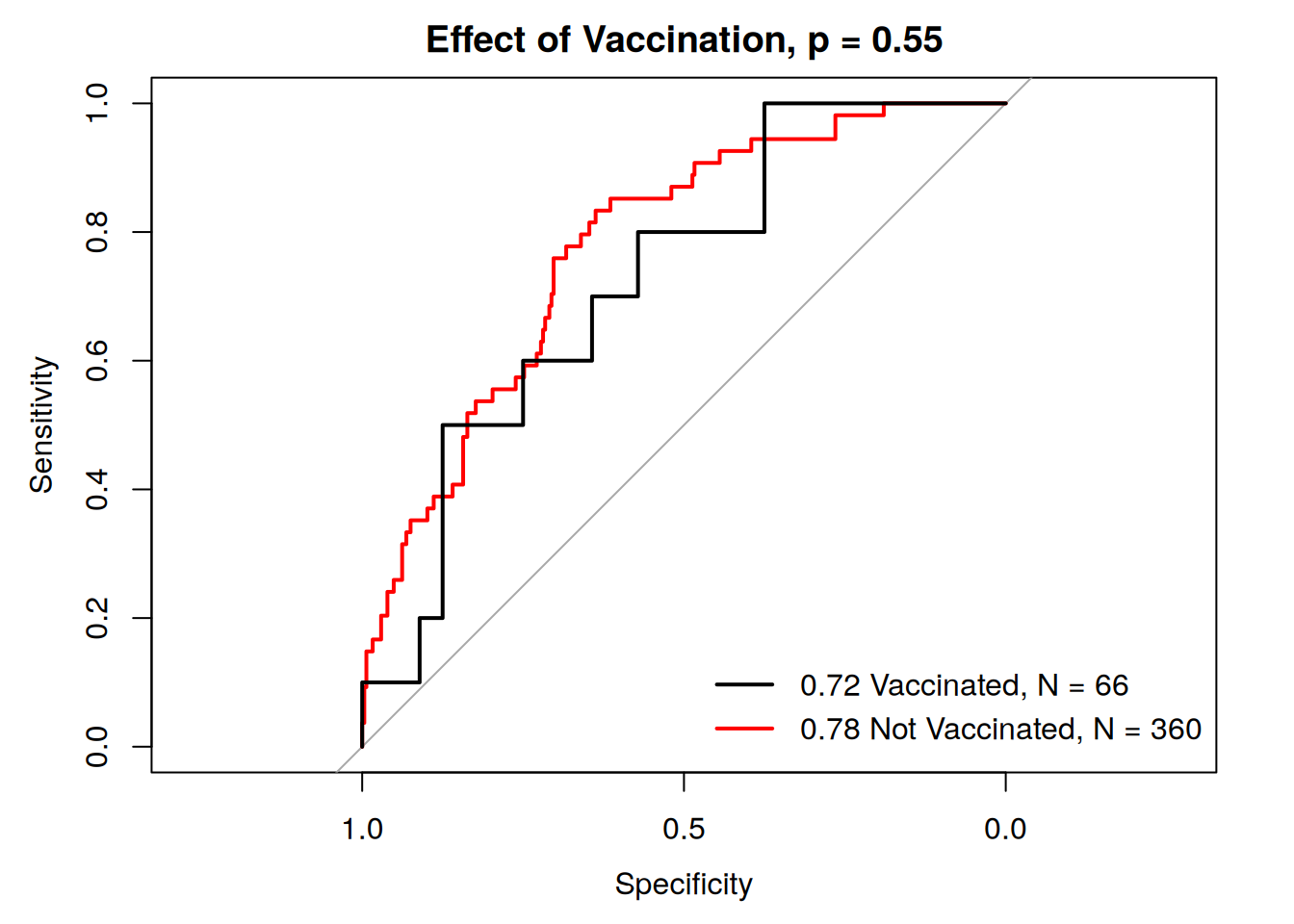
**Fig. S11:** Comparison of prediction performances to assess the impact of vaccination against COVID-19.

**Supplementary Tables:**

**Table S1:** Net benefit of ET-1 alone for different risk thresholds

| **Risk threshold** | **Cost-benefit ratio** | **% Patients**  **above threshold** | **net benefit**  **(EP ~ ET-1)** | **Standardized net benefit** |
| --- | --- | --- | --- | --- |
| 0.05 | 1:19 | 78 | 0.11 | 0.76 |
| 0.1 | 1:9 | 57 | 0.08 | 0.55 |
| 0.15 | 3:17 | 37 | 0.05 | 0.37 |
| 0.2 | 1:4 | 27 | 0.04 | 0.26 |
| 0.25 | 1:3 | 17 | 0.03 | 0.15 |
| 0.3 | 3:7 | 12 | 0.02 | 0.11 |
| 0.35 | 7:13 | 9 | 0.01 | 0.07 |
| 0.4 | 2:3 | 7 | 0.01 | 0.05 |

**Table S2:** Comparison of performance of prediction models

|  | **Endpoint** | **Prediction Models** | | |
| --- | --- | --- | --- | --- |
|  |  | **CRB-65 + TnT-hs + PCT**  **(AUC)** | **CRB-65 + ET-1**  **(AUC)** | **p-value** |
| **COVID-19** | 28d mortality | 0.90  (95% CI: 0.85-0.95) | 0.82  (95% CI: 0.73-0.91) | 0.01 |
|  | ICU admission | 0.63  (95% CI: 0.55-0.70) | 0.75  (95% CI: 0.69-0.81) | 0.15 |
| **Non-COVID CAP** | 28d mortality | 0.85  (95% CI: 0.77-0.91) | 0.88  (95% CI: 0.81-0.96) | 0.20 |

AUC: area under the ROC Curve

References

van Calster, B., McLernon, D. J., van Smeden, M., Wynants, L., & Steyerberg, E. W. (2019). Calibration: The Achilles heel of predictive analytics. *BMC Medicine*, *17*(1), 230. https://doi.org/10.1186/s12916-019-1466-7
